# Event-Related Potential-Based Brain–Computer Interface Using the Thai Vowels’ and Numerals’ Auditory Stimulus Pattern

**DOI:** 10.3390/s22155864

**Published:** 2022-08-05

**Authors:** Manorot Borirakarawin, Yunyong Punsawad

**Affiliations:** 1 School of Informatics, Walailak University, Nakhon Si Thammarat 80160, Thailand; 2 Informatics Innovative Center of Excellence, Walailak University, Nakhon Si Thammarat 80160, Thailand

**Keywords:** brain–computer interface, electroencephalography, event-related potential (ERP), auditory stimulation

## Abstract

Herein, we developed an auditory stimulus pattern for an event-related potential (ERP)-based brain–computer interface (BCI) system to improve control and communication in quadriplegia with visual impairment. Auditory stimulus paradigms for multicommand electroencephalogram (EEG)-based BCIs and audio stimulus patterns were examined. With the proposed auditory stimulation, using the selected Thai vowel, similar to the English vowel, and Thai numeral sounds, as simple target recognition, we explored the ERPs’ response and classification efficiency from the suggested EEG channels. We also investigated the use of single and multi-loudspeakers for auditory stimuli. Four commands were created using the proposed paradigm. The experimental paradigm was designed to observe ERP responses and verify the proposed auditory stimulus pattern. The conventional classification method produced four commands using the proposed auditory stimulus pattern. The results established that the proposed auditory stimulation with 20 to 30 trials of stream stimuli could produce a prominent ERP response from Pz channels. The vowel stimuli could achieve higher accuracy than the proposed numeral stimuli for two auditory stimuli intervals (100 and 250 ms). Additionally, multi-loudspeaker patterns through vowel and numeral sound stimulation provided an accuracy greater than 85% of the average accuracy. Thus, the proposed auditory stimulation patterns can be implemented as a real-time BCI system to aid in the daily activities of quadratic patients with visual and tactile impairments. In future, practical use of the auditory ERP-based BCI system will be demonstrated and verified in an actual scenario.

## 1. Introduction

Practical brain–computer interface (BCI) systems for medical applications are complicated. Design and implementation techniques are required to build a communication system between the brain and external devices or systems without muscular activities [1,2,3,4]. A BCI-based assistive technology for controlling external devices or machines for mobility enhancement and rehabilitation has been proposed previously [5]. Varying disability levels can pose problems for developing BCI systems. Previous research has focused on user efficiency for evaluating the cognition function in their proposed BCI systems. Electroencephalography (EEG) is a popular technique for acquiring brain data for non-invasive BCI systems [4,5,6]. Currently, EEG devices are rapidly being developed for clinical [7,8,9] and non-clinical applications, such as entertainment [10,11], transport [12], and education [13], such as in an examination and comparison of the EEG-based attention test with continuous performance test (CPT) and test of variables of attention (TOVA) to mention the relationship between a cognitive psychological attention test and the attention levels [14]. Moreover, applications of brainwave-based control to examine the effect of different web-based media on human brain waves by BCI systems might increase knowledge transfer efficiency in digital educational materials [15].

Visual evoked potential (VEP) is a widely exploited EEG signal generated by the visual cortex in response to visual stimulation. VEP-based BCI is a high-performance BCI method [16,17,18]. The method has been widely implemented to enhance communication and control; its applications include BCI spellers based on transient VEP (or P300) [19] and steady-state VEP (SSVEP)-based BCI for machine control [20,21]. Moreover, a low-cost human–machine interface system can also support some patients via techniques such as eye-tracking [22]. Such systems require normal visual functions. Moreover, somatosensory evoked potential-based BCI based on tactile stimulation has been proposed for patients with impaired vision or eye movements and hearing [23,24].

In cases of amyotrophic lateral sclerosis (ALS) or locked-in syndrome (LIS) patients with visual and tactile impairment, auditory stimulation is an alternative method of brain activation, and modulation generating evoked potentials (EPs) can be measured using EEG. Event-related potentials (ERPs) are brain responses that involve sensory, cognitive, or motor events. ERP-based BCI usually employs external sound, light, and tactile stimulation. Similar to the VEP-based BCI, an auditory evoked potential (AEP)-based BCI [25] can be categorized according to the stimulus method and EEG changes into two types: (1) auditory ERPs, which are transient responses [26], and (2) steady-state AEP-BCI [27]. AEP-based BCIs require selective auditory attention based on the human hearing process. Neurology can reveal the pathway between both the auditory nerve and cortex in the perception of encoding and auditory processing inputs [28]. Furthermore, auditory recognition and memory processes have been reported previously [29]. Auditory stimuli are popular techniques for evaluation and treatment processes in psychology and neurotherapy [7,30].

In previous research on AEP-based BCIs, as shown in Table 1, researchers mainly considered protocols and paradigms of auditory stimulation methods and the position of electrodes. Most studies have demonstrated that EEG signals obtained from the frontal, temporal, and parietal areas produce an evoked response (P300) that synchronizes with auditory tasks. For auditory stimulation, Pokorny et al. [31] provided different sound tones (low tone: 396 Hz; high tone: 1900 Hz) for the left and right sides of the stimulus using earphones to create more than a single command in the auditory BCI system. The system achieved up to 90.6% accuracy. Moreover, Cai et al. [32] implemented eight sound stimuli directions with two different short white and pink noise stimuli bursts through eight loudspeakers in an octagonal setting for a multi-command BCI. An offline experiment reported that the proposed auditory stimulus paradigm improved the results by providing higher classification results and an improved information transfer rate than conventional paradigms.

Voice, as auditory stimuli, may be easy to recognize in a multi-command BCI. Matsumoto et al. [33] proposed the use of English vowel sounds such as “a”, “e”, “i”, “o”, and “u” through inner ear headphones for auditory stimulation. The results showed that the accuracy ranged from 60 to 100%. In addition, Halder et al. [34] adopted the Japanese Hiragana syllabary using earphones for a multi-command BCI. Their proposed BCI system demonstrated an average accuracy of over 70%. In addition, Onishi et al. [35] employed a positive affective sound (a meowing cat sound) and negative affective sound (a screaming cat sound) with the answers “yes” and “no”. The results indicated that the affective auditory BCI system yielded a high classification accuracy. Furthermore, Simon et al. [36] demonstrated an auditory BCI for spellers using natural stimuli from five animal voices, i.e., duck, bird, frog, seagull, and dove. The results indicated that the training session increased the efficiency of healthy subjects and patients with ALS.

A steady-state auditory response (ASSR) is usually utilized to enhance the auditory ERP responses in a BCI, and the stimulation can yield high-intensity EEG features in the frequency domain. Kaongoen and Jo [37] demonstrated the combination of ASSR and P300 paradigms for a hybrid-auditory BCI. Different sound modulations for the left and right sides, such as a 1 kHz sound with an amplitude modulation (AM) frequency of 37 Hz and a 2 kHz sound with an AM frequency of 43 Hz, can increase the difference between the sound sources in the auditory P300-based BCI system. The system produces superior classification results.

According to previous research, as shown in Table 1, auditory BCIs can be used as an alternative for developing assistive devices. In this study, we attempted to improve the performance of an AEP-based BCI system for control and communication among patients with severe paralysis. However, the sound-stimulus pattern for the proposed auditory stimulus paradigm is challenging for a practical auditory BCI [38]. Hence, we suggested and observed the auditory ERP stimulation paradigm by employing Thai vowels with a pronunciation similar to that of English vowels for a multicommand auditory P300-based BCI system [33,35,39]. Furthermore, Thai numeral sounds were included to improve classification accuracies. We additionally verified several stimulus trials and sound durations to reduce the command-creation time and improve the performance of the ERP components. Overall, this study addresses two primary issues: (1) verification of auditory ERP stimulation patterns using different sounds and loudspeaker position settings with the proposed experimental paradigm and different number of trials; and (2) observation of the differences in the ERP responses obtained from the proposed stimulus pattern between the use of vowel (Previous studies) and numeral sounds (Proposed) for real-time auditory BCI systems. The proposed methods were implemented on healthy participants and will be further investigated and compared with other BCI techniques.

## 2. Materials and Methods

### 2.1. EEG Signal Acquisition

Twelve healthy volunteers, seven females and five males (average age of 27 ± 3.6 years), participated in the ERP experiments. All of the selected subjects had normal hearing acuity and no signs of brain disorders, mental disorders, auditory nerve symptoms, or migraines (past or present). Subjects with aural neurological complications or hearing loss of >40 dB were excluded. After reading the documentation, all participants provided written informed consent. All signed consent forms, without personal identification, were kept confidential. All experiments involving human subjects were approved by the Office of the Human Research Ethics Committee of Walailak University (WU-EC-EN-2-140-64, 14 June 2021), which acknowledged the Ethical Declaration of Helsinki, Council for International Organizations of Medical Sciences, and World Health Organization guidelines.

**Figure 1:**
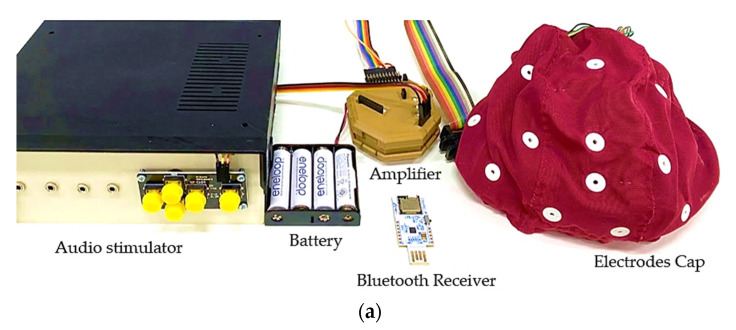

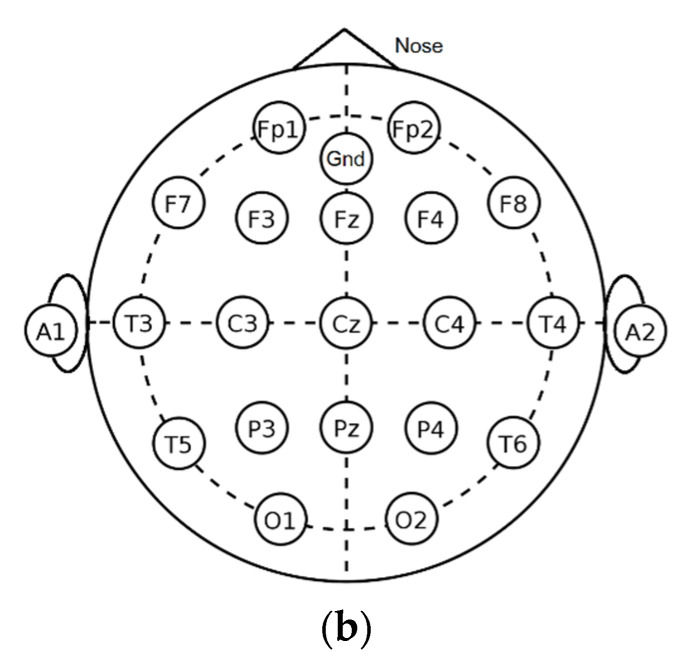
(**a**) Components of the proposed auditory BCI system. (**b**) Electrode placement for 19 channels based on the 10–20 system.

The components of the auditory BCI system (Figure 1a) consisted of an OpenBCI with an eight-channel EEG amplifier used to acquire an EEG signal using cap electrodes. Eight EEG electrodes were positioned at Fp1, Fz, Cz, T3, T4, Pz, P3, and P4, following the 10–20 system (Figure 1b) to cover the auditory cortex, frontal, and brain midline areas related to the task requirements, based on previous studies [31,32,33,34]. The reference electrode was at the A1 position. The EEGs were recorded at a sampling rate of 256 Hz using the OpenBCI software. The audio stimulator with speakers was connected to the OpenBCI system for marking the EEG with different sound stimuli. MATLAB (MathWorks) [ver. R2019a] was used to process and analyze the recorded EEG signals from each subject. A 50 Hz notch filter was used to remove power line noises, and a 0.1 Hz to 30 Hz bandpass digital filter was used for motion artifact removal via the EEGLAB toolbox [40]. Subsequently, we employed the ERPLAB toolbox to segment EEG signals and detect ERP responses [41].

### 2.2. Proposed Auditory Stimulation Patterns and Paradigm

**Figure 2:**
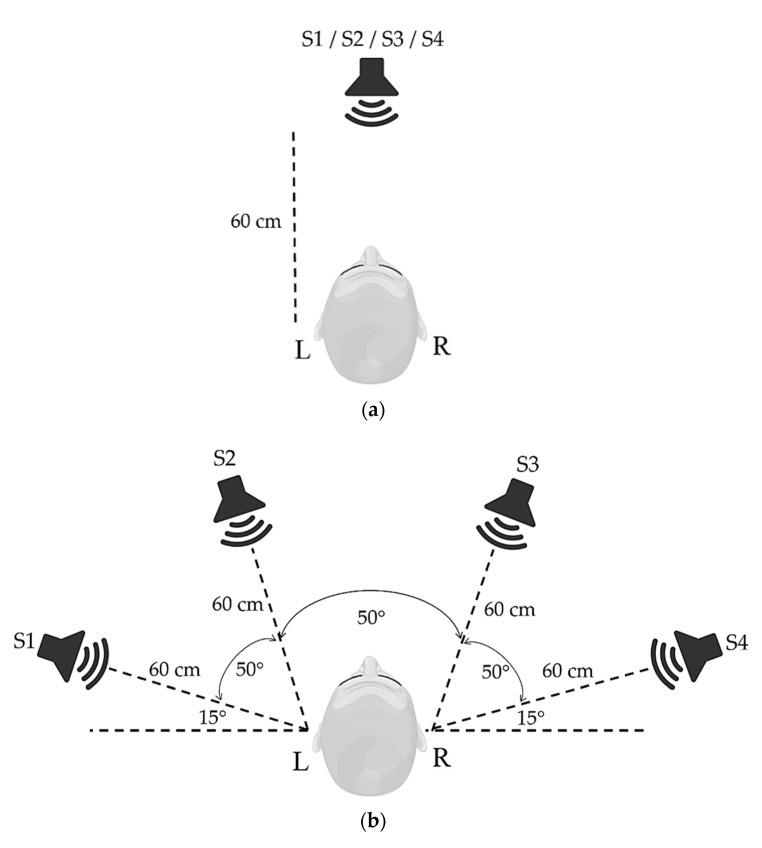
Two patterns of the loudspeaker position settings for auditory stimulation. (**a**) Single-loudspeaker position setting. (**b**) Four loudspeaker position settings in front of the participant.

For a practical auditory BCI, loudspeakers are used for auditory stimulation, such as inherent hearing, to observe the proposed sound stimulus [32]. Similarly, we also propose an auditory stimulus paradigm for BCI systems. According to previous studies [33,34,35,36], this research focuses on applying the auditory BCI to Thai people and then to people belonging to other nationalities. Auditory stimulus intervals (ASI) as tone duration and trials of auditory stimulations using the four vowels “a”, “e”, “i”, and “o”, because their sounds are similar to those of some Thai vowels. Moreover, Thai numeral sounds that were easy to recognize were used on all participants. We performed a pilot study to validate Thai numerals with various durations of ASI through reaction time (RT) testing. Most participants reported unclear hearing for durations < 100 ms, affecting sound recognition. Therefore, we compressed each sound file into 100 ms (minimum duration that can be recognized) and 250 ms (normal duration) durations to observe the ERP responses and features for setting the sound intervals. The audio stimulator included an Arduino Uno microcontroller board that cooperated with a mini MP3 module to generate a random sound pattern stimulation (sound level: 70 dB), and it sent trigger signals to the OpenBCI board, as shown in Figure 1a. Two patterns of the loudspeaker position settings were developed. The loudspeakers were located in front of the participant. The distance between the loudspeaker and the participant was 60 cm for each pattern, following the human spatial auditory performance and the loudspeaker specification (Figure 2). The loudspeakers were placed in the same plane as that of the participant’s ears. The first pattern used only a single loudspeaker, as shown in Figure 2a, for steaming four auditory stimuli, as shown in Table 2. The second pattern was employed apart from sound sources using four loudspeakers set in a semicircle for spatial auditory stimuli [32,42,43], as shown in Figure 2a.

**Figure 3:**
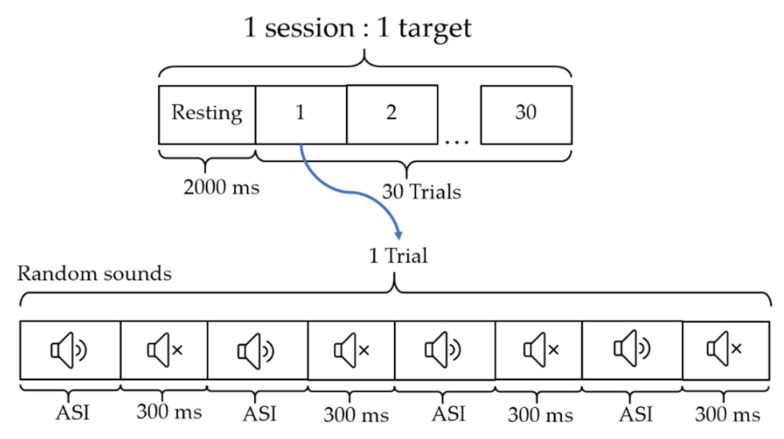
Experimental paradigm of the auditory stimulus to investigate the proposed auditory stimuli and the pattern of loudspeaker position settings. (Auditory stimulus intervals (ASI): 100 and 250 ms).

The experimental setup is illustrated in Figure 3. The paradigm is set to start in a resting state for baseline collection at 2000 ms. A single session consisting of 30 trials of an auditory stream stimulus with two ASI, that is, 100 ms and 250 ms, was used for verification. Subsequently, the sound was muted for 300 ms. Each trial consisted of four random stimulus sounds. The participant performed only the target sounds following the sequence in Table 3 for vowel sounds and Table 4 for numeral sounds as 12 targets (sessions) for three rounds per sound (vowel and numeral) with two proposed auditory stimulation patterns (single and multi-loudspeaker). Before proceeding to the new round, participants were allowed to rest for 5 min.

### 2.3. Auditory ERP Detections

According to previous studies presented in Table 1, the ERP components, such as N200 (N2) and P300 (P3), were used for EEG feature extraction and as parameters. Note that N200 and P300 exhibit negative voltage peaks and positive voltage values, respectively, with latencies of 150–250 and 250–500 ms, respectively, after the onset of the stimulus. For each EEG channel, the stimulus data were segmented at intervals of 200 ms before the onset and at 600 ms after the onset to collect the baseline and ERP components, respectively. The average was calculated for each trial to predict the target for each stimulation pattern and participant.

Stepwise linear discriminant analysis (SWLDA) employs a combination of forward and backward stepwise analyses of variable features. SWLDA can provide robust feature extraction and a good prediction for P300-based BCIs [44]. The most correlated features are selected to predict the targeted command based on statistical significance [45]. The conventional technique using N200 and P300 features and SWLDA can yield a high efficiency for both offline [31,32,33] and online testing [35,36]. The training ERP dataset was used to generate the weight of the discriminant model. The SWLDA process starts without feature parameters in the discriminant model. The forward analysis enters an input feature parameter into the model. The model is evaluated by an F-test. If the *p*-value < 0.01, the variable is added. The backward step removes the least significant feature parameter (*p* < 0.015). Thus, linear regression is used to include or exclude feature parameters for prediction. The test ERP dataset was computed with the feature weights to distinguish the target and determine a maximum score for classification [36,45].

### 2.4. Experiments

**Figure 4:**
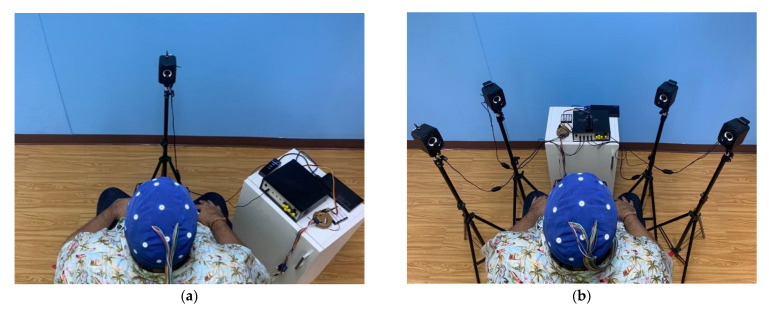
Experimental setup of the auditory stimuli evoked EEG potentials. (**a**) Auditory stimulus with a single loudspeaker. (**b**) Auditory stimulus with multi loudspeakers.

Before EEG recording, we had to prepare the participants by placing the cap electrode and checking the position of the electrode by following the 10–20 system (Figure 1b). The experiment was conducted in a quiet room with typical indoor lighting. The participants sat in front of the loudspeaker, as shown in Figure 4. Then, participants received instructions on the auditory stimulus experiment paradigm shown in Figure 3. Each participant attended the target sound, as shown in Table 3 and Table 4. The experimental setup is illustrated in Figure 4. The classification accuracies were determined as the number of correct output targets to validate the proposed auditory ERP stimulus pattern.

## 3. Results

### 3.1. Observation Corresponding to the Use of Sound of Numerals for Auditory ERP Stimulation

**Figure 5:**
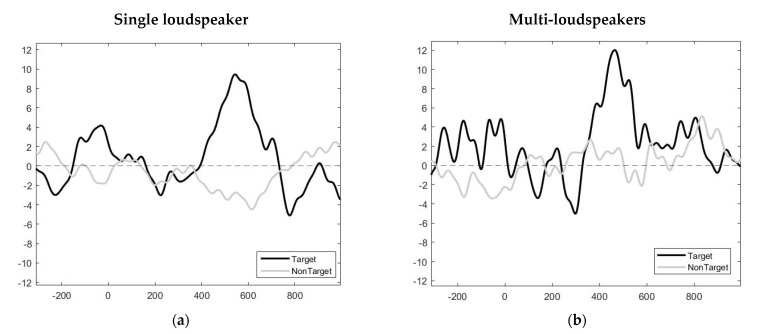
Example of grand−averaged ERPs obtained from all subjects over the auditory stimulus interval 250 ms. (**a**) Grand−averaged ERPs from the auditory stimulus based on the sounds of numerals produced via a single loudspeaker. (**b**) Grand−averaged ERPs from the auditory stimulus based on the sound of numerals produced via multiple loudspeakers.

Before using the sound of numerals, we had to observe ERP features by creating the grand-averaged EPR for visualization using the EEGLAB toolbox, as shown in Figure 5. The black lines show the target responses, and the gray lines show the non-target responses. We can demonstrate filtered EEG data obtained from the subject showing a clear P300 in the black plot that occurred approximately 400 ms after the onset of the stimuli. Peaks can be observed at approximately 200 ms and 500 ms for one speaker and multiple speakers, respectively. The results revealed that the ERP components were similar to those of the previous auditory ERP-based BCI.

### 3.2. Observation Corresponding to the Use of EEG Electrodes for ERP Detection

**Figure 6:**
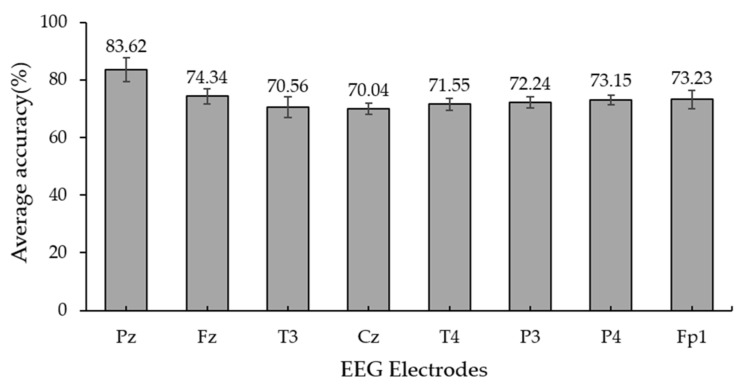
Average classification accuracy corresponding to different positions of electrodes of the proposed auditory ERP stimulation using SWLDA. Confidence interval (Alpha: 0.05).

According to Figure 6, we selected the Cz, Fz, and Pz electrodes to obtain additional responses from parietal cortices known to generate ERPs related to spatial and P300 responses. This figure presents the verification of the electrodes for ERP detection. The maximum average accuracy of the electrode was 83.62% for Pz. We observed that the EEG from channel Pz exhibited a positive peak for the target sound. We can observe a grand−averaged ERP in Figure 5. This channel was used to create parameters for classification using a minimum N200 response combined with a maximum P300 response.

### 3.3. Verification of the Proposed Auditory ERP Stimulus Pattern

#### 3.3.1. Auditory Stimulus Trials and Intervals

**Figure 7:**
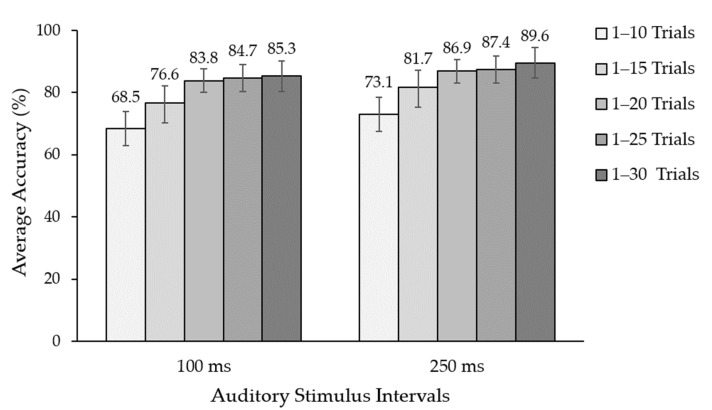
Average classification accuracy between 100 ms and 250 ms in the auditory stimulus intervals. Confidence interval (Alpha: 0.01).

The trial number and stimulus intervals are essential parameters for the efficiency of auditory BCIs, such as the information transfer rate (ITR). Therefore, we also validated the number of trials for each ASI for the real-time auditory ERP BCI. The EEG recorded from 30 trials was divided into five ranges for ERP detection. Figure 7 shows that the average classification accuracy ranged from 65.5% to 89.6%, and the average classification accuracy in the number of trials was 20, 25, and 30 at an ASI of 100 ms, and for an ASI of 250 ms, the average classification accuracy was more than 80%. The ASI of 250 ms achieved more than 85% of the average classification accuracy. We observed a maximum classification accuracy of 89.6% for the ASI at 250 ms in 30 trials. The ASI at 100 ms with 20 trials reached an average classification accuracy of 83.8% and 32 s for generating an output faster than the ASI at 250 ms, which used 44 s. Moreover, the ASI at 250 ms, with 15 trials yielded approximately 80% of the average classification accuracy using 33 s for output creation. We employed 20 and 25 trials to observe an ASI with different sounds, such as vowels and numerals.

According to Table 5, the average classification accuracy of the auditory stimulus with an ASI at 100 ms ranged from 69.4% to 92.5%. The average classification accuracy for the ASI at 250 ms ranged from 77.5% to 90.5%. The maximum classification accuracy of the sound stimulus of numerals was 88.5%, whereas the maximum classification accuracy of the sound stimulus of vowels was 92.5% for the ASI at 100 ms. In addition, the average accuracy of the numeral stimuli with the ASI at 250 ms was 83.8%, which was greater than the average accuracy of the ASI at 100 ms. The results suggested that the sound stimulus of vowels with the ASI at 100 ms can produce a high average classification accuracy for all participants, as opposed to the sound stimulus of numerals.

**Figure 8:**
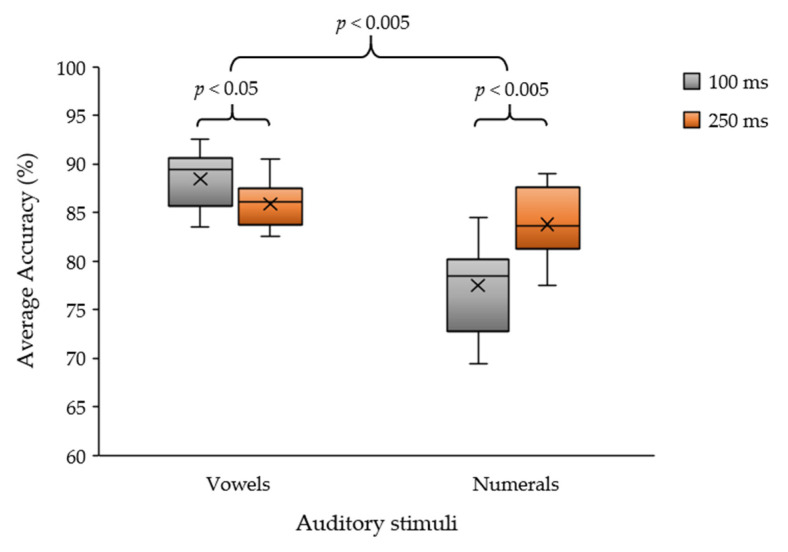
Average classification accuracy between vowels and numeral sounds in auditory stimulus intervals of 100 ms and 250 ms.

We observed a significant difference between 100 ms and 250 ms in the stimulus intervals of vowel and numeral (acquainted sound) stimuli. We used the paired *t*-test to analyze statistically significant differences between the stimulus intervals and sound stimuli groups. The results are shown in Figure 8. We found that the paired *t*-test (*n* = 12) indicated that there was a significant difference in the average classification accuracy between the stimulus intervals of 100 and 250 ms for the vowels (*p* = 0.024; *p* < 0.05). Figure 8 also indicates a significant difference between the accuracies obtained for the stimulus intervals of 100 and 250 ms for numeral stimuli (*p* = 0.004; *p* < 0.005). Moreover, a statistically significant difference could be identified between the average classification accuracy obtained from the vowel and numeral stimuli, irrespective of the stimulus intervals (*p* = 0.000; *p* < 0.005).

#### 3.3.2. Auditory Stimulation Patterns

According to the results in Table 6, the average accuracy of the vowel auditory stimuli ranged from 71.0% to 97.5% for all subjects and that for the numerical stimuli ranged from 67.5% to 95.0%. We found that both sounds provided similar efficiencies. The second test is an evaluation of the number of speakers between single and multiple (four) loudspeakers for comparison between vowel and numeral sound stimuli. The average accuracy of using the vowel sound was 86.0% and the numerical sound was 75.1% for a single loudspeaker. The average accuracy of the vowel sound was 87.2% and that of numerals was 86.2% for multiple loudspeakers, which was greater than the average accuracy of the single speaker. These results verified and supported the proposed auditory stimulus pattern. The vowel and numerical patterns obtained with the multi-loudspeaker method can generate the average classification accuracy of all subjects. For some participants, both stimulus patterns yielded similar classification accuracies for each auditory stimulation-detection method.

**Figure 9:**
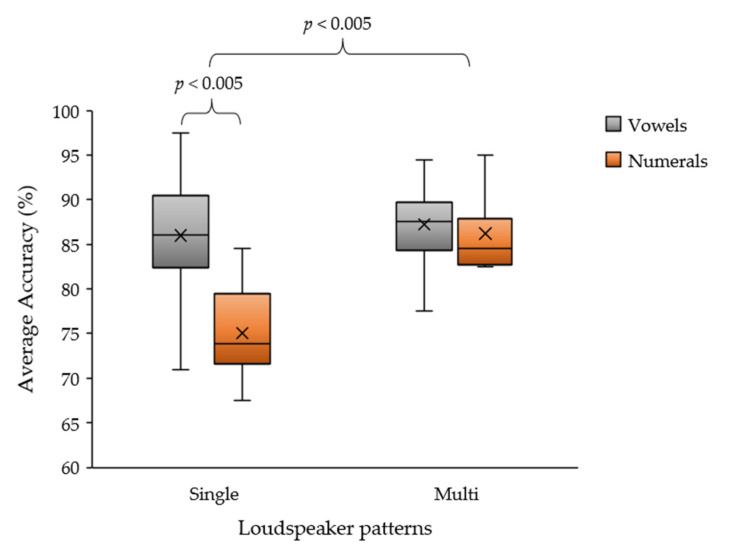
Average classification accuracy between vowel and numeral sounds of the auditory ERP stimulus of single and multi-loudspeaker patterns.

According to Figure 9, two main issues were observed. The first issue concerns the efficiency of each loudspeaker pattern. The paired *t*-test (*n* = 24) showed a statistically significant difference between the single- and multiple-loudspeaker patterns (*p* = 0.003; *p* < 0.005). In addition, using four loudspeakers could yield a higher efficiency than using a single loudspeaker. The second issue is the efficiency of each auditory stimulus. The paired *t*-test (*n* = 12) indicated a significant difference between the vowel and numeral auditory stimuli using a single loudspeaker (*p* = 0.001; *p* < 0.005). Furthermore, the paired t-test indicated no significant difference between the vowel and numeral patterns using the multi-loudspeaker method (*p* = 0.559; *p* > 0.05). Even though the proposed auditory stimulus via a numeral pattern can achieve a higher average classification accuracy than the vowel pattern for all subjects, fatigue with concentration on sound must be observed between the vowel and numeral patterns for practical purposes.

## 4. Discussion

Observing the proposed auditory ERP stimuli via a numeral stimulus (in Thai) for the auditory BCIs, ERP responses of the numeral stimulus pattern showed obvious N200 and P300 responses, refs. [31,36] which were mainly produced by the mid-frontal and mid-parietal areas (Fz and Pz). Moreover, we verified auditory stimulus intervals and trials for ERP detection using the SWLDA method. The experiment reported that 100 ms of ASI with more than 20 stimulus trials and 250 ms of ASI with more than 15 stimulus trials achieved acceptable accuracies. In addition, the ASI at 100 ms of the vowel stimulus provided a better ERP response than the numeral stimulus. The 250 ms numeral (word) stimulation yielded acceptable accuracies. Furthermore, we investigated the efficiency of single- and multi-loudspeakers position patterns of auditory stimuli. The experiment reported that spatial auditory stimuli using multi-loudspeaker yielded better classification accuracy than using only a single loudspeaker for vowel and numeral sound streaming.

Nevertheless, some limitations of the proposed auditory ERP stimulus patterns for BCI are as follows. (1) Users require a training session for recognition, and they need to be guided on how to pay attention to target sounds. (2) The proposed method requires a long time to create each command, which results in a low ITR. This disadvantage should be addressed for real-time BCI systems. The addition of a command to enable/disable an auditory stimulator is recommended for practical use. In addition, the consideration of further validation can be listed as follows: (1) the sound level and distance between loudspeakers require a recommendation protocol; (2) longer intervals can be applied after successive stimuli for studying the cognitive components; (3) a usability test can be performed to compare related BCI techniques such as tactile BCI [24]; (4) a greater number of targets can be employed to verify the system efficiency and user workload.

## 5. Conclusions

This paper proposes an auditory ERP pattern for a practical BCI system for quadriplegia with visual impairments. We verified the auditory stimulus pattern to observe the ERP responses to Thai vowel and numeral sounds with different stimulus intervals and a number of trials for command classification. We found that a vowel auditory stimulus interval of 100 ms for 20 trials (32 s per command) and a numeral auditory stimulus interval of 250 ms for 15 trials (33 s per command) can be used for an auditory BCI. In addition, the loudspeaker positions and patterns were investigated. As reported previously [40,41,42], the proposed multi-loudspeaker pattern via a spatial auditory stimulation can yield a higher classification accuracy than a single loudspeaker for both vowel and numeral stimuli. Finally, the proposed auditory stimulation can be used in real-time BCI systems for quadratic patients with visual and tactile impairments. We suggested that the multi-loudspeaker patterns for auditory stimuli via vowel and numeral sounds can be implemented in auditory ERP-based BCI systems for control and communication to aid in their daily activities. In future studies, we will verify the practical use of the auditory ERP stimulation with an independent loudspeaker position in actual situations with natural noise.

## Figures and Tables

**Table 1:** Research studies on the auditory stimulus for event related potential (ERP)-based brain–computer interface (BCI).

Author	Proposed Method	Auditory Stimuli	Speakers	Electrodes	Result(s)
Pokorny et al., 2013 [31]	The auditory P300-based single-switch brain–computer interface	- Low tones (396 Hz) and the deviants (297 Hz) in 300 ms - High tones (1900 Hz), and the deviants (2640 Hz) in 600 ms	Earphones	F3, Fz, F4, C3, Cz, C4, P3, Pz, P4	The mean classification accuracies lie between 69% and 83.5% for a stepwise linear discriminant analysis classifier for 12 Minimally conscious state patients
Cai et al., 2015 [32]	Spatial Auditory Brain–Computer Interface	The white- and pink-noise stimuli of 30 ms lengths with 5 ms	Eight loud speakers	P3, P4, P5, P6, Cz, CPz, Pz, POz	The proposed model can boost the results by up to +10.43 bit/min (+44% classification accuracy).
Matsumoto et al., 2013 [33]	P300 Responses to Various Vowel Stimuli for an Auditory BCI	Vowels stimuli “a”, “e”, “i”, “o”, and “u” duration was set to 100 ms	Inner ear headphones	Cz, CPz, POz, Pz, P1, P2, C3, C4, O1, O2, T7, T8, P3, P4, F3, F4	Classification accuracies between 60% and 100%
Halder et al., 2016 [34]	Auditory P300 BCIs via Hiragana syllabary	46 Hiragana characters with a two-step procedure: First the consonant (ten choices) and then the vowel (five choices)	Earphones	AF7, FPz, AF8, F3, Fz, F4, C3, Cz, C4, CP3, CPz, CP4, P4, Pz, P4, POz	The mean classification accuracy is above 70%
Onishi et al., 2017 [35]	Affective Stimuli for an Auditory P300 BCI	Positive and negative affective sounds (PA: a meowing cat sound, NA: a screaming cat sound)	Earphones	C3, Cz, C4, P3, Pz, P4, O1, O2	ALS patients achieved 90% online classification accuracy
Simon et al., 2015 [36]	An auditory multi-class BCI for a speller	Natural stimuli from five animal voices i.e., duck, bird, frog, seagull, and dove. Each tone had a length of 150 ms and ISI of 250 ms.	Headphones	F3, Fz, F4, C5, C3, C1, Cz, C2, C4, C6, CP5, CP3, CP1, CPz, CP2, CP4, CP6, P3, P1, Pz, P2, P4, PO7, PO3, POz, PO4, PO8, Oz.	Healthy participants achieved an average accuracy of 90%. The average accuracy of ALS patients was 47%.
Kaongoen & Jo, 2017 [37]	ASSR/P300-hybrid auditory BCI system	Two sounds were set to have 1 kHz and 2 kHz pitches with 37 Hz and 43 Hz AM frequencies, respectively	Earphones	Fz, Cz, T3, T4, Pz, P3, P4, Oz	The maximum average classification accuracy for each setting are P300: 98.38%, and ASSR: 85.42%

**Table 2:** Vowel and numeral sounds for auditory-evoked potential stimulation.

Sound Stimulus	Vowels		Numerals	
	Thai	English	Thai	English
**S1**	เอ	a	หนึ่ง	neung
**S2**	อี	e	สอง	søng
**S3**	ไอ	i	สาม	sām
**S4**	โอ	o	สี่	sī

**Table 3:** Target of the auditory ERP components using stimuli as vowel sounds with a duration of 100 and 250 ms.

Session	1	2	3	4	5	6	7	8	9	10	11	12
**Target**	a	i	e	o	i	i	o	e	e	i	o	a

**Table 4:** Target of the auditory ERP components using stimuli as numeral sounds with a duration of 100 and 250 ms.

Session	1	2	3	4	5	6	7	8	9	10	11	12
**Target**	neung	sām	søng	sī	sām	neung	sī	søng	søng	sām	sī	neung

**Table 5:** Results of the average classification accuracy of different stimuli intervals (tone duration) of vowels and numerals for each participant.

Auditory Stimuli	Average Classification Accuracy (%)			
	Vowels		Numerals	
Participants	ASI: 100 ms	ASI: 250 ms	ASI: 100 ms	ASI: 250 ms
1	88.8	87.5	80.0	88.5
2	90.3	86.3	84.5	88.0
3	90.0	84.8	79.5	84.5
4	90.8	86.0	72.5	82.5
5	85.0	83.8	73.5	81.7
6	92.5	86.5	77.5	85.0
7	90.0	90.5	77.5	87.5
8	87.5	87.5	72.5	77.5
9	91.5	82.5	80.0	82.8
10	85.4	88.9	69.4	89.0
11	83.5	83.5	82.5	78.5
12	86.5	82.5	80.3	80.0
Mean ± SD.	88.5 ± 2.86	85.8 ± 2.53	77.5 ± 4.57	83.8 ± 3.95

**Table 6:** Results of the average classification accuracy under different loudspeaker patterns of vowel and numeral stimuli for each participant.

Loudspeaker Patterns	Average Classification Accuracy (%)			
	Single		Multi	
Participants	Vowels	Numerals	Vowels	Numerals
1	87.5	75.0	88.8	93.5
2	82.8	84.5	93.8	88.0
3	93.5	80.0	81.3	84.0
4	82.3	72.5	94.5	82.5
5	85.0	71.7	83.8	83.5
6	91.5	80.0	87.5	82.5
7	97.5	70.0	90.0	95.0
8	87.5	67.5	87.5	82.5
9	86.5	77.8	87.5	85.0
10	85.5	71.5	88.8	86.9
11	71.0	77.5	86.0	83.5
12	81.5	72.8	77.5	87.5
Mean ± SD.	86.0 ± 6.71	75.1 ± 4.95	87.2 ± 4.79	86.2 ± 4.24

## Data Availability

The data presented in this study are available upon reasonable request.
